# The Impact of the Menopausal Transition on Body Composition and Abdominal Fat Redistribution

**DOI:** 10.3390/jcm15020740

**Published:** 2026-01-16

**Authors:** Anna Szeliga, Peter Chedraui, Blazej Meczekalski

**Affiliations:** 1 Department of Gynecological Endocrinology, Poznan University of Medical Sciences, 61-701 Poznan, Poland; 2 Escuela de Postgrado en Salud & Centro de Investigaciones, Universidad Espíritu Santo, Samborondón 092301, Ecuador; peterchedraui@outlook.com

**Keywords:** menopausal transition, body composition, lean mass and visceral fat, sarcopenic obesity

## Abstract

**Objective:** To evaluate the impact of the menopausal transition on body composition across different body mass categories and to identify menopause-related changes in lean and fat tissue distribution. **Methods:** This retrospective cross-sectional study included 325 women whose clinical and body composition data were extracted from existing records. Participants were classified as premenopausal (controls), perimenopausal, or postmenopausal and further stratified by body mass index (BMI) into normal-weight, overweight, and obesity groups. Body composition had been assessed using bioelectrical impedance analysis. **Results:** Across all BMI categories, postmenopausal women demonstrated significantly lower lean body mass, soft lean mass, skeletal muscle mass, total body water, protein, and mineral content compared with premenopausal and perimenopausal women (*p* < 0.05). Total and visceral fat area (VFA), body fat percentage (BF), and waist-to-hip ratio were significantly higher, indicating a shift toward central adiposity. These changes were most pronounced in normal-weight women (VFA: 36.4 ± 17.0, 48.3 ± 22.3, and 55.7 ± 23.5 cm^2^; BF: 24.8 ± 5.3%, 27.2 ± 5.2%, and 28.8 ± 4.6% in pre-, peri-, and postmenopause, respectively), and less marked among overweight women (VFA: 91.5 ± 36.3, 106.1 ± 38.2, and 111.7 ± 28.6 cm^2^; BF: 36.0 ± 3.6%, 36.4 ± 3.9%, and 37.2 ± 3.2%) and with obesity (VFA: 180.3 ± 62.4, 212.6 ± 96.2, and 175.5 ± 54.4 cm^2^; BF: 44.5 ± 4.5%, 44.5 ± 5.7%, and 41.9 ± 3.3%), suggesting a relative attenuation of muscle loss at higher BMI. **Conclusions:** Postmenopausal women showed a clear shift toward lower lean mass and greater central adiposity across BMI categories. These patterns indicate a consistent deterioration in body composition during the menopausal transition. Assessment of visceral fat in postmenopausal women is crucial, as its accumulation is closely linked to cardiometabolic risk. Menopause-related hormonal changes favor central adiposity, supporting the use of visceral fat as a key indicator for early risk stratification and preventive interventions in midlife women.

## 1. Introduction

Early research indicates that the menopausal transition is associated with unfavorable shifts in body fat distribution, particularly an increase in abdominal adiposity, which elevates cardiovascular risk [1]. Estrogen plays a central role in regulating body fat distribution and white adipose tissue health. Its bioavailability promotes the accumulation of metabolically healthy subcutaneous fat rather than visceral fat and protects against metabolic dysfunction [2].

Beyond shaping fat distribution, ovarian estrogen also confers protection against obesogenic factors and metabolic diseases. Declines in circulating estrogen—whether due to menopause or ovariectomy—are linked to increased risks of obesity, type 2 diabetes, and cardiovascular disease. In both humans and animal models, estradiol replacement therapy or hormone replacement therapy (HRT) counteracts obesity by reducing visceral fat mass and improving metabolic health [2].

Estrogen deficiency during the perimenopausal period contributes to increased fat tissue mass and reduced lean tissue mass [3]. Hormone therapy with estrogen and progestogen in early postmenopausal women may help prevent these changes by reducing fat accumulation and preserving lean mass [3]. The endocrine alterations associated with menopause further amplify the effects of body weight and fat distribution. These hormonal shifts promote visceral fat deposition and may explain the higher incidence of cardiovascular disease in postmenopausal women [4].

Previous studies have shown that postmenopausal women tend to exhibit higher total and visceral fat and lower lean mass compared with premenopausal women; however, findings remain inconsistent due to differences in study design, age disparities between comparison groups, and limited consideration of body mass index (BMI) and metabolic status. While abdominal fat redistribution across the menopausal transition has been relatively well described, less is known about concurrent changes in skeletal muscle mass and other body composition compartments, including body water, protein, and mineral content, particularly among women with overweight or obesity. Importantly, the clinical implications of these compositional changes—especially their relevance to cardiometabolic risk and sarcopenic obesity—remain insufficiently characterized in routine clinical practice. Therefore, this study aims to address this gap by providing a detailed assessment of body composition across pre-, peri-, and postmenopausal stages, stratified by BMI, to improve understanding of menopause-related changes that may inform early risk identification and targeted preventive strategies for preserving muscle health and reducing adverse metabolic outcomes in midlife women.

### Study Objective

The aim of this study was to evaluate the impact of the menopausal transition on body composition in women across different body mass categories and to identify menopause-related changes in lean and fat tissue distribution.

## 2. Materials and Methods

This retrospective study included 325 healthy women hospitalized at the Department of Gynecological Endocrinology, Gynecology, and Obstetrics at the University Hospital of the Poznań University of Medical Sciences between 1 January and 30 April 2022. Participants were recruited using convenience sampling, based on availability during hospitalization and fulfillment of predefined inclusion criteria. Women were classified into three groups based on menopausal status: premenopausal, perimenopausal, and postmenopausal. The perimenopausal group comprised 58 women (29 normal weight, 16 overweight, and 13 with obesity). Inclusion criteria for this group were: age 45–55 years, regular or irregular menstrual cycles, follicle-stimulating hormone (FSH) levels of 15–25 mIU/mL, and estradiol (E2) levels > 100 pg/mL. The postmenopausal group included 50 women (19 normal weight, 16 overweight, and 15 with obesity). Inclusion criteria for this group were: at least one year since the last menstrual period, FSH levels > 25 mIU/mL, and E2 levels < 10 pg/mL.

Within each menopausal group, women were further stratified according to body mass index (BMI) into the following categories: normal weight (18.5–24.9 kg/m^2^), overweight (25.0–29.9 kg/m^2^), and obesity (30.0–39.9 kg/m^2^) categories.

The control (premenopausal) group consisted of 217 healthy women, including 126 with normal weight, 47 who were overweight, and 44 with obesity. Eligible participants were required to have regular menstrual cycles, defined as menses occurring at consistent intervals of 27–35 days for at least six consecutive months prior to enrollment, based on medical history. Inclusion criteria further required the absence of hormonal abnormalities, as confirmed by screening hormonal and metabolic profiles, and no evidence of kidney or liver disease, as assessed by hematocrit, complete blood count, aspartate aminotransferase (AST), alanine aminotransferase (ALT), and creatinine measurements, as well as pelvic examination and anamnesis. A eugonadal ovulatory status was confirmed by serum progesterone concentrations > 5 ng/mL during the luteal phase of the menstrual cycle, corresponding to the time of examination.

Exclusion criteria, applied uniformly across all study groups, included the use of hormonal therapy at the time of examination or within the preceding six months, the presence of severe chronic disease, with particular emphasis on cardiovascular disease, and any history of oncological treatment. Fifty-six women who initially met the age and hormonal inclusion criteria were subsequently excluded due to comorbid conditions and/or the use of medications that could affect body composition.

The study was conducted in accordance with the principles of the Declaration of Helsinki. The research protocol was reviewed and approved by the Bioethical Committee of the Poznań University of Medical Sciences (approval No. 125/23). Informed consent was not required due to the retrospective nature of the study.

Body composition was assessed in all participants using the ACCUNIQ BC380 bioelectrical impedance analyzer (SELVAS Healthcare, Daejeon, Republic of Korea). This device provides non-invasive measurements of fat mass, lean mass, and other body composition parameters. For each participant, the following parameters were evaluated: body composition, hormonal profile, lipid profile, glucose, and insulin levels.

All body composition measurements were performed in the morning under fasting conditions. Participants were instructed to refrain from food and fluid intake for 8–10 h prior to assessment to minimize hydration-related variability, which can influence BIA-derived estimates.

For each patient included in the study, venous blood was drawn from the antecubital vein via temporary venous access into a dry plastic tube in the morning hours (between 6:00 and 10:00 a.m.). Prior to blood collection, all patients fasted for 8–10 h. After collection, the blood samples were centrifuged at 1500 *g* for 10 min to obtain serum.

In the control group, blood was drawn during the follicular phase of the menstrual cycle, approximately on days 11–13, when a dominant follicle (≥10 mm in diameter) was present in the ovary and serum estradiol concentration exceeded 90 pg/mL.

Serum analyses for both the study and control groups were performed at the Central Laboratory of the Gynecology and Obstetrics University Hospital, Poznań University of Medical Sciences. Concentrations of FSH, luteinizing hormone (LH), E2, prolactin, testosterone, and dehydroepiandrosterone sulfate (DHEA-S) were measured in morning samples (6:00–10:00 a.m.). Thyroid function was also assessed—thyroid-stimulating hormone (TSH) and free thyroxine (fT4)—to exclude thyroid disorders.

Hormone concentrations were determined using the electrochemiluminescence immunoassay (ECLIA) method on a Cobas e601 analyzer (Roche Diagnostics International, Rotkreuz, Switzerland).

Parameters of metabolic balance, including fasting glucose and insulin levels, were assessed. The homeostasis model assessment of insulin resistance (HOMA-IR) index was calculated using the formula: HOMA-IR = fasting insulin (µU/mL) × fasting glucose (mmol/L)/22.

A complete lipid profile was obtained, including total cholesterol (TC), high-density lipoprotein cholesterol (HDL-C), low-density lipoprotein cholesterol (LDL-C), and triglycerides (TG). TC, HDL-C, LDL-C, and TG concentrations were measured using the enzymatic–colorimetric method. Insulin concentrations were determined using the electrochemiluminescence immunoassay (ECLIA) method, and fasting glucose was measured using the reference enzymatic hexokinase method.

### Statistical Analysis

All analyses were performed using STATISTICA Version 12 (StatSoft, 2012). Data distribution was assessed using the Shapiro–Wilk test. For comparisons involving three or more groups according to menopausal status and BMI categories, one-way analysis of variance (ANOVA) was applied for normally distributed variables, and the Kruskal–Wallis test was used for non-normally distributed variables. When a significant overall group effect was identified, post hoc pairwise comparisons were performed with appropriate correction for multiple testing.

Associations between continuous variables were assessed using Spearman’s rank-order correlation. A two-sided *p*-value < 0.05 was considered statistically significant.

## 3. Results

Anthropometric, hormonal, lipid, and carbohydrate metabolism data are summarized in Table 1. The study group consisted of 58 perimenopausal women (29 normal weight, 16 overweight, and 13 with obesity). The postmenopausal group included 50 women (19 normal weight, 16 overweight, and 15 with obesity), while the control (premenopausal) group consisted of 217 healthy women, including 126 with normal weight, 47 who were overweight, and 44 with obesity.

Compared with premenopausal women, peri- and postmenopausal women showed significant differences in FSH, LH, E2, DHEA-S, and testosterone, while lipid profile, insulin, glucose, and thyroid hormones did not differ significantly.

Body composition results are presented in Table 2. Among normal-weight participants, postmenopausal women had lower lean body mass (*p* = 0.01), soft lean mass (*p* = 0.01), total body water (*p* = 0.01), protein (*p* = 0.01), mineral content (*p* < 0.01), and skeletal muscle mass (*p* < 0.01) compared with premenopausal and perimenopausal women. They also had higher body fat percentage (*p* < 0.01), BMI (*p* < 0.01; within normal range), waist-to-hip ratio (*p* < 0.01), and visceral fat area (*p* < 0.01). Perimenopausal women displayed higher body fat mass than controls (*p* = 0.03).

In the overweight group, postmenopausal women had a higher waist-to-hip ratio (*p* < 0.01) and visceral fat area (*p* = 0.02) than controls. In the obesity group, postmenopausal women showed reduced body fat mass compared with controls (*p* = 0.04).

Fasting glucose in the normal-weight group was higher in perimenopausal women than in controls (*p* = 0.002). In the overweight group, postmenopausal women had higher fasting glucose compared to both controls (*p* < 0.001) and perimenopausal women (*p* = 0.022), with total cholesterol also elevated in postmenopausal women (*p* = 0.025). In the obesity group, postmenopausal women showed higher fasting glucose (*p* = 0.008), higher total cholesterol (*p* = 0.013), and increased insulin levels compared with perimenopausal women (*p* = 0.037).

## 4. Discussion

### 4.1. Body Composition Changes Across the Menopausal Transition

Before menopause, fat and lean mass rise gradually. With the menopausal transition, fat accumulation roughly doubles and lean mass declines, stabilizing about two years after the final menstrual period; body weight rises steadily but plateaus thereafter [5]. The present study shows that postmenopausal women have lower lean body mass, skeletal muscle, total body water, protein, and minerals, alongside higher total and visceral fat, body fat percentage, and waist-to-hip ratio, reflecting central adiposity. Estrogen decline drives these changes. Women with overweight/obesity lose muscle less rapidly, but greater fat deposition offsets this, increasing metabolic risk.

Panotopoulos et al. [6] studied women with obesity, and, despite adjusting trunk fat for total fat mass, it remains unclear whether observed patterns reflect menopause or obesity. Postmenopausal women with obesity (n = 73) had more fat in the trunk and less fat and lean mass in femoral and leg regions than premenopausal women, with perimenopausal women showing similar shifts. Ley et al. [7] reported that postmenopausal women (n = 70) had 20% higher total fat than premenopausal women (n = 61, *p* < 0.001), with more android fat (42.1% vs. 38.3%, *p* < 0.001) and less gynoid fat, confirming a postmenopausal shift toward central adiposity. These studies reinforce menopause-associated fat gain and redistribution.

Postmenopausal women have lower bone and lean mass but higher fat mass than premenopausal women [4]. These changes occur without differences in total body weight, showing that menopause alters body composition proportions—reducing bone and lean tissue while increasing fat—rather than overall weight [4]. Our results align with these findings.

Many studies include postmenopausal women who are considerably older than premenopausal participants, making it difficult to separate menopausal from age-related effects [1]. While abdominal fat increases with years since menopause, this is not independent of age [3]. Lean tissue loss may reflect aging, sedentary behavior, lower protein intake, and reduced energy expenditure, which may also contribute to fat gain. Exercise and calorie restriction in postmenopausal women and older adults reduce fat and increase lean mass, supporting the role of lifestyle and aging alongside hormonal changes [3].

Evidence from a large cohort shows that the menopausal transition is associated with reductions in lean body mass, lean body mass index, appendicular lean mass, appendicular lean mass index, leg lean mass, and thigh muscle cross-sectional area [8]. Menopausal status strongly predicts these losses, while physical activity helps preserve lean and appendicular muscle mass, mitigating declines across multiple body regions [8].

### 4.2. Mechanisms: The Role of Estrogen Deficiency

Declining estradiol during the menopausal transition predicts reductions in lean and appendicular mass and thigh muscle cross-sectional area, reflecting accelerated muscle loss [9], consistent with the decreases observed in the present study. Reduced estradiol and elevated FSH are associated with visceral fat gain and muscle catabolism, partly through increased proinflammatory cytokines that promote protein breakdown and suppress anabolic pathways. These processes contribute to sarcopenic obesity and elevate chronic disease and mortality risk [9].

Our study found that postmenopausal women had lower lean body mass, soft lean mass, protein content, and skeletal muscle mass compared with pre- and perimenopausal women. Skeletal muscle contains estrogen receptors that regulate satellite cell activation, proliferation, and fiber regeneration. Declining estrogen during menopause impairs muscle maintenance and repair and shifts fat from peripheral subcutaneous depots, especially gluteofemoral, toward central visceral stores, reinforcing the postmenopausal pattern of increased abdominal adiposity [9].

### 4.3. Consequences of Muscle Loss for Women’s Health

Muscle loss during and after menopause has major health implications. Reduced skeletal muscle decreases strength and physical performance, increasing frailty, falls, and risk of functional dependence. Lower muscle mass also reduces resting energy expenditure, promoting fat gain and metabolic imbalance, and impairs glucose uptake, predisposing to insulin resistance and metabolic syndrome. Additionally, muscle loss compromises bone density and increases osteoporosis risk, as contractions and mechanical loading are vital for bone integrity. Collectively, these changes can diminish quality of life and accelerate age-related decline [10,11,12]. In line with this, we observed that glucose metabolism was altered in peri- and postmenopausal women, particularly those with overweight or obesity.

### 4.4. Bone and Muscle Interactions

Early postmenopausal bone loss is linked to estrogen deficiency and concurrent body composition shifts. During menopause, fat mass increases while lean mass declines, changes that may continue for up to two years after the final menstrual period [5]. Body composition influences bone through mechanical loading, physical activity, and nutritional, genetic, and hormonal factors [13].

Skeletal muscle supports bone via mechanical forces and paracrine/endocrine factors that regulate remodeling. Adipose tissue has dual effects: it can enhance bone mineral density (BMD) through loading and androgen-to-estrogen conversion but also produces proinflammatory cytokines that stimulate osteoclasts and reduce BMD [13].

Studies on body composition and BMD during menopause are mostly cross-sectional, with inconsistent findings; some show positive correlations between lean mass and BMD, while results for fat mass vary [13].

### 4.5. Cardiometabolic Implications and Sarcopenic Obesity

Menopause-related hormonal changes amplify the effects of body weight and fat distribution on skeletal and metabolic health. Endocrine alterations promote visceral fat accumulation, which worsens bone loss through systemic inflammation and disrupts hormonal balance, while diminishing the mechanical benefits of higher body weight [14].

Abdominal adipose tissue secretes proinflammatory cytokines and adipokines that negatively affect bone and metabolism, highlighting the importance of distinguishing visceral from subcutaneous fat [14].

Postmenopausal women exhibit central (android) fat distribution, reduced abdominal lipolysis, and increased femoral lipoprotein lipase activity [3]. This pattern is linked to insulin resistance, hyperinsulinemia, and unfavorable lipid changes, increasing cardiovascular risk after menopause [4]. In our study, peri- and postmenopausal women—particularly those with overweight or obesity—showed altered lipid profiles, reinforcing these mechanistic links.

Visceral obesity triggers chronic inflammation via immune activation, raising TNF, IL-6, and leptin while IGF-1 declines, promoting insulin resistance, muscle catabolism, and fat gain. Increased leptin and reduced adiponectin further impair muscle growth and fat oxidation, driving sarcopenic obesity. These interconnected mechanisms create a vicious cycle that magnifies menopause-related body composition changes [9].

### 4.6. Methodological Considerations (BIA vs. DXA)

Although bioelectrical impedance analysis (BIA) is practical and widely used, its accuracy can be affected by hydration and the prediction equations applied for different devices and populations, leading to variable performance [15]. Comparative studies show that BIA generally aligns with Dual-energy X-ray Absorptiometry (DXA) at the group level, but systematic bias and wide individual limits of agreement may occur, potentially causing misclassification. Therefore, our findings should be interpreted with caution [9].

Conversely, DXA-derived trunk fat estimates have limitations. While menopause is consistently associated with increased central adiposity, DXA cannot precisely locate fat within the trunk, and intra-abdominal fat is particularly relevant for estimating cardiovascular and diabetes risk [16].

BIA-derived estimates of visceral fat are indirect and may be influenced by hydration status and the prediction algorithms used. While BIA correlates reasonably well with DXA and CT at the population level, it lacks the anatomical precision of imaging-based methods, particularly for intra-abdominal fat compartments. Therefore, visceral fat values in this study should be interpreted primarily as relative indicators rather than absolute measurements.

Future research may benefit from calibration against DXA or CT or reporting device-specific error metrics according to consensus recommendations [9]. BIA’s non-invasive, rapid, radiation-free, cost-effective, and portable nature supports routine use, especially for repeated measures, large-scale screening, and longitudinal monitoring [9,17,18,19].

### 4.7. Study Limitations, Future Perspectives, and Final Remark

This retrospective cross-sectional study limits causal inference between menopause, hormonal changes, and body composition alterations. Although groups were matched by BMI, differences in age and lifestyle may have influenced results. Future longitudinal studies should include direct measures of muscle quality, inflammatory markers, and hormonal profiles to clarify the temporal relationship between menopause and lean and fat mass changes. Interventions with physical activity, resistance training, and adequate protein intake may help mitigate muscle loss and metabolic risk during and after menopause.

Importantly, many studies, including the present one, compare postmenopausal women who are substantially older than premenopausal controls, which limits the ability to disentangle menopause-related hormonal effects from those of chronological aging. Although abdominal fat accumulation increases with years since menopause, this process is not independent of age-related changes in body composition, physical activity, and energy expenditure. Accordingly, the observed differences should be interpreted as menopause-associated patterns rather than menopause-specific effects, particularly within a cross-sectional framework.

Additionally, an important limitation of this study is the lack of data on lifestyle-related factors such as physical activity, dietary intake, and protein consumption. These variables are known to strongly influence skeletal muscle mass, fat distribution, and metabolic health, particularly during the perimenopausal and postmenopausal periods, and may have contributed to the observed between-group differences. Moreover, the analyses were not adjusted using multivariable regression models that included age or metabolic parameters. Consequently, residual confounding cannot be excluded, and the observed associations between menopausal status and body composition may partly reflect age-related or metabolic influences rather than menopause-specific effects.

## 5. Conclusions

Postmenopausal women were characterized by lower lean mass and a greater degree of central adiposity across BMI categories. These findings indicate a consistent association between menopausal stage and adverse body composition patterns. Routine assessment of skeletal muscle and visceral fat may help identify women at increased cardiometabolic risk during midlife, supporting early preventive strategies. Targeted preventive strategies should include lifestyle interventions such as regular resistance and weight-bearing exercise, optimization of protein intake, and promotion of physical activity aimed at preserving muscle mass and limiting visceral fat accumulation. Early implementation of these measures may help mitigate cardiometabolic risk, reduce the development of sarcopenic obesity in women who develop obesity, and preserve functional capacity and quality of life in midlife women.

## Figures and Tables

**Table 1:** Anthropometric data, results of hormonal, lipid profile, and carbohydrate metabolism in divided groups of patients.

	Premenopausal			Perimenopausal			Postmenopausal		
Parameters	Normal	Overweight	Obesity	Normal	Overweight	Obesity	Normal	Overweight	Obesity
Age (years)	28.59 ± 8.12 (27.15–30.02)	31.11 ± 8.50 (28.61–33.60)	30.98 ± 7.18 (28.79–33.16)	45.97 ± 4.99 (44.07–47.87)	46.44 ± 2.80 (44.94–47.93)	46.69 ± 4.13 (44.19–49.19)	53.56 ± 5.39 (50.87–56.24)	50.6 ± 13.87 (42.92–58.28)	55.18 ± 8.48 (49.48–60.88)
FSH (mIU/mL)	6.63 ± 2.59 (6.2–7.1)	5.93 ± 2.03 (5.33–6.52)	6.2 ± 2.38 (5.48–6.93)	22.29 ± 17.74 (15.54–29.04)	18.87 ± 18.50 (9.01–28.73)	15.93 ± 9.71 (10.06–21.80)	95.14 ± 40.22 (75.14–115.14)	71.49 ± 19.47 (60.70–82.27)	65.10 ± 30.75 (44.44–85.76)
LH (mIU/mL)	13.61 ± 16.05 (10.8–16.4)	10.89 ± 9.95 (7.97–13.82)	12.04 ± 13.37 (7.97–16.10)	20.38 ± 12.89 (15.47–25.28)	22.0 ± 17.98 (12.42–31.58)	14.67 ± 13.17 (6.70–22.63)	42.84 ± 18.35 (33.72–51.97)	36.49 ± 10.65 (30.59–42.39)	34.49 ± 9.47 (28.12–40.86)
E2 (pg/mL)	151.54 ± 126.70 (129.2–173.8)	148.24 ± 102.07 (118.27–178.21)	135.93 ± 92.82 (107.71–164.15)	168.82 ± 146.64 (113.04–224.60)	263.62 ± 323.30 (91.34–435.89)	128.09 ± 118.19 (56.66–199.51)	6.59 ± 5.73 (3.74–9.43)	18.36 ± 31.95 (0.66–36.05)	10.24 ± 8.16 (4.76–15.73)
Prolactin (ng/mL)	23.03 ± 93.48 (6.5–39.6)	16.02 ± 7.61 (13.78–18.26)	14.68 ± 6.22 (12.79–16.57)	12.09 ± 4.71 (10.29–13.88)	9.32 ± 5.73 (6.14–12.49)	13.96 ± 6.27 (10.17–17.76)	18.49 ± 38.52 (−0.66–37.65)	10.92 ± 3.90 (8.75–13.08)	8.36 ± 3.40 (6.08–10.67)
Testosterone (ng/mL)	0.42 ± 0.18 (0.37–0.45)	0.45 ± 0.20 (0.391–0.51)	0.44 ± 0.23 (0.37–0.51)	0.28 ± 0.20 (0.19–0.36)	0.31 ± 0.13 (0.24–0.38)	0.23 ± 0.13 (0.14–0.31)	0.14 ± 0.11 (0.08–0.19)	0.16 ± 0.12 (0.09–0.23)	0.19 ± 0.09 (0.12–0.26)
DHEA-S (µmol/L)	7.78 ± 3.56 (7.15–8.41)	8.55 ± 3.72 (7.45–9.64)	7.75 ± 3.88 (6.57–8.94)	5.06 ± 2.49 (4.11–6.01)	4.89 ± 1.39 (4.14–5.63)	4.21 ± 2.37 (2.78–5.65)	3.51 ± 2.42 (2.31–4.71)	3.95 ± 2.09 (2.79–5.12)	3.08 ± 1.72 (1.91–4.24)
TSH (µIU/mL)	2.20 ± 1.23 (1.98–2.42)	2.11 ± 1.23 (1.75–2.48)	2.00 ± 1.20 (1.64–2.37)	2.03 ± 0.99 (1.65–2.41)	1.73 ± 0.92 (1.24–2.22)	2.90 ± 3.29 (0.90–4.89)	2.27 ± 2.1 (1.22–3.32)	2.41 ± 1.78 (1.43–3.40)	2.34 ± 1.77 (1.15–3.54)
fT4 (ng/mL)	1.29 ± 0.31 (1.24–1.35)	1.31 ± 0.27 (1.23–1.39)	1.29 ± 0.29 (1.20–1.38)	1.28 ± 0.14 (1.19–1.30)	1.27 ± 0.14 (1.19–1.35)	1.25 ± 0.20 (1.13–1.38)	1.25 ± 0.16 (1.17–1.33)	1.19 ± 0.11 (1.12–1.25)	1.27 ± 0.15 (1.17–1.37)
Insulin (µU/mL)	8.32 ± 3.33 (7.73–8.91)	10.73 ± 4.91 (9.28–12.17)	20.93 ± 22.03 (14.23–27.63)	7.29 ± 2.70 (6.26–8.32)	10.52 ± 5.03 (7.84–13.21)	14.9 ± 7.2 (10.52–19.24)	9.04 ± 3.76 (7.17–10.91)	11.53 ± 6.62 (7.86–15.20)	18 ± 3.5 (15.63–20.31)
Glucose (mg/dL)	94.97 ± 10.49 (93.09–96.79)	96.68 ± 6.66 (94.70–98.66)	103.4 ± 8.8 (100.76–106.09)	99.66 ± 7.09 (96.97–102.36)	98.69 ± 9.6 (93.57–103.80)	114.03 ± 31.56 (94.96–133.10)	96.96 ± 5.06 (94.44–99.47)	107.43 ± 10.56 (101.58–113.27)	114.7 ± 18.4 (102.35–127.03)
Non-HDL-C (mg/dL)	129.84 ± 32.11 (47.01–77.31)	118.42 ± 44.24 (92.88–143.96)	134.94 ± 31.06 (125.26–144.61)	132.04 ± 14.69 (113.79–150.29)	145.46 ± 36.64 (114.83–176.09)	140.71 ± 25.75 (125.14–156.27)	153.42 ± 34.41 (132.63–174.21)	146.87 ± 29.51 (124.18–169.55)	159.64 ± 33.21 (135.88–183.39)
Cholesterol (mg/dL)	192 ± 39.20 (143.32–240.68)	175.1 ± 45 (148.99–201.02)	185.5 ± 32.4 (175.43–195.60)	208.78 ± 22.88 (180.36–237.19)	203.85 ± 29.82 (178.91–228.79)	200.56 ± 30.69 (182.01–219.10)	221.13 ± 35.12 (199.91–242.35)	212.3 ± 21.3 (195.92–228.61)	216 ± 35.1 (190.92–241.12)
HDL-C (mg/dL)	62.16 ± 12.20 (47.01–77.31)	56.59 ± 11.48 (49.95–63.22)	50.58 ± 10.50 (47.31–53.86)	76.74 ± 12.25 (61.53–91.95)	58.39 ± 16.15 (44.88–71.89)	59.85 ± 17.21 (49.44–70.25)	67.71 ± 35.12 (59.08–76.33)	65.4 ± 14.91 (53.93–76.87)	56.38 ± 12.72 (47.28–65.48)
LDL-C (mg/dL)	128.8 ± 29.42 (92.27–165.33)	110.89 ± 44.80 (85.02–136.75)	126.24 ± 31.15 (116.53–135.94)	129.34 ± 15.96 (109.51–149.17)	130.54 ± 30.00 (105.45–155.62)	132.45 ± 31.99 (113.12–151.79)	161.22 ± 30.91 (142.54–179.89)	141.11 ± 25.43 (121.56–160.66)	145.25 ± 33.59 (121.22–169.28)
Triglyceride (mg/dL)	75.74 ± 18.84 (52.34–99.14)	81.5625.42 (66.89–96.24)	120.99 ± 50.70 (105.19–36.79)	102.34 ± 42.01 (50.17–154.51)	127.8 ± 64.06 (74.24–181.36)	120.41 ± 48.53 (91.08–149.74)	88.16 ± 40.07 (63.95–112.37)	133.01 ± 66.79 (81.67–184.36)	147.56 ± 62.61 (102.77–192.35)

Data are presented as mean ± standard deviations and 95% confidence intervals in parentheses.

**Table 2:** Body composition assessment in divided groups.

	Premenopausal			Perimenopausal			Postmenopausal		
Parameters	Normal	Overweight	Obesity	Normal	Overweight	Obesity	Normal	Overweight	Obesity
Lean body mass [kg]	44.2 ± 5.6 (43.26–45.21)	47.5 ± 5.1 (45.98–48.95)	54.2 ± 6.5 (52.22–56.19)	45.1 ± 5.8 (42.97–47.35)	46.2 ± 4.8 (43.65–48.81)	53.1 ± 6.9 (48.85–57.21)	41.1 ± 3.5 (39.38–42.88)	46.1 ± 4.8 (43.38–48.64)	52.4 ± 5.9 (48.34–56.40)
Soft lean mass [kg]	41.0 ± 5.2 (40.13–41.96)	44.1 ± 4.7 (42.67–45.44)	50.3 ± 6.1 (48.44–52.14)	41.9 ± 5.4 (39.92–43.99)	42.9 ± 4.5 (40.55–45.34)	49.3 ± 6.5 (45.37–53.18)	38.2 ± 3.3 (36.53–39.82)	42.8 ± 4.5 (40.28–45.24)	48.7 ± 5.6 (44.93–52.39)
Total body water [L]	32.4 ± 4.1 (31.64–33.08)	34.7 ± 3.7 (33.62–35.79)	39.6 ± 4.8 (38.13–41.03)	33.1 ± 4.2 (31.49–34.68)	33.9 ± 3.5 (31.96–35.74)	38.8 ± 5.1 (35.75–41.88)	30.1 ± 2.6 (28.82–31.39)	33.7 ± 3.5 (31.76–35.64)	38.3 ± 4.3 (35.42–41.25)
Proteins [kg]	8.7 ± 1.1 (8.49–8.89)	9.3 ± 1.0 (9.04–9.64)	10.7 ± 1.3 (10.30–11.09)	8.9 ± 1.2 (8.44–9.33)	9.1 ± 0.9 (8.57–9.61)	10.4 ± 1.4 (9.61–11.29)	8.1 ± 0.7 (7.71–8.42)	9.5 ± 0.9 (8.52–9.58)	10.3 ± 1.2 (9.52–11.12)
Minerals [kg]	3.2 ± 0.4 (3.11–3.29)	3.4 ± 0.4 (3.30–3.52)	3.9 ± 0.5 (3.77–4.05)	3.2 ± 0.4 (3.05–3.36)	3.3 ± 0.4 (3.08–3.47)	3.8 ± 0.5 (3.49–4.04)	2.95 ± 0.2 (2.83–3.07)	3.26 ± 0.3 (3.08–3.44)	3.7 ± 0.5 (3.38–4.00)
Mass of body fat [kg]	14.9 ± 4.6 (14.09–15.71)	26.7 ± 3.8 (25.62–27.82)	43.9 ± 9.4 (41.07–46.75)	16.8 ± 3.6 (15.48–18.21)	26.4 ± 3.4 (24.61–28.24)	43.5 ± 13.1 (35.56–51.39)	16.8 ± 3.6 (15.00–18.62)	27.1 ± 2.8 (25.49–28.69)	38.1 ± 6.5 (33.74–42.44)
Skeletal muscle mass [kg]	24.6 ± 3.1 (24.08–25.17)	26.4 ± 2.8 (25.60–27.27)	30.2 ± 3.6 (29.06–31.28)	25.2 ± 3.2 (23.94–26.39)	25.8 ± 2.7 (24.32–27.20)	29.6 ± 3.9 (27.24–31.89)	22.9 ± 1.9 (21.92–23.89)	25.6 ± 2.7 (24.16–27.13)	29.2 ± 3.3 (26.97–31.43)
Body mass index [kg/m^2^]	21.2 ± 2.3 (20.8–21.6)	27.4 ± 1.6 (26.92–27.86)	35.7 ± 4.3 (34.45–37.03)	22.7 ± 1.5 (22.15–23.26)	27.5 ± 1.1 (26.87–28.06)	35.4 ± 6.1 (31.69–39.02)	22.1 ± 1.8 (21.19–23.08)	27.3 ± 1.2 (26.66–28.02)	33.3 ± 2.5 (31.62–34.94)
Percent body fat [%]	24.8 ± 5.3 (23.88–25.76)	36.0 ± 3.6 (34.96–37.05)	44.5 ± 4.5 (43.10–45.87)	27.2 ± 5.2 (25.23–29.17)	36.4 ± 3.9 (34.33–38.47)	44.5 ± 5.7 (41.01–47.88)	28.8 ± 4.6 (26.52–31.14)	37.2 ± 3.2 (35.33–38.89)	41.9 ± 3.3 (39.76–44.20)
Waist-to-Hip Ratio	0.76 ± 0.05 (0.75–0.77)	0.86 ± 0.05 (0.85–0.88)	0.9 ± 0.1 (0.95–0.98)	0.8 ± 0.05 (0.78–0.81)	0.9 ± 0.1 (0.86–0.91)	1.0 ± 0.1 (0.95–1.04)	0.8 ± 0.05 (0.80–0.85)	0.89 ± 0.04 (0.87–0.92)	0.9 ± 0.1 (0.94–1.01)
Visceral fat area [cm^2^]	36.4 ± 17.0 (33.42–39.42)	91.53 ± 36.28 (80.88–102.19)	180.3 ± 62.4 (161.30–199.24)	48.3 ± 22.3 (39.79–56.77)	106.1 ± 38.2 (85.77–126.48)	212.6 ± 96.2 (154.49–270.73)	55.7 ± 23.5 (44.11–67.44)	111.67 ± 28.55 (95.85–127.48)	175.5 ± 54.4 (138.98–212.11)

Data are presented as mean ± standard deviations and 95% confidence intervals in parentheses.

## Data Availability

The data presented in this study are available by contacting the corresponding authors.
